# Adolescents' perceptions of the credibility of informational content on fitness and dietary supplements: The impact of banner and native advertising

**DOI:** 10.1002/jad.12394

**Published:** 2024-08-20

**Authors:** David Lacko, Hana Machackova, Lukáš Slavík

**Affiliations:** ^1^ Interdisciplinary Research Team on Internet and Society, Faculty of Social Studies Masaryk University Brno Czechia; ^2^ Department of Media Studies and Journalism, Faculty of Social Studies Masaryk University Brno Czechia

**Keywords:** adolescents, advertisements, banner advertising, credibility, fitness/dietary supplements, native advertising

## Abstract

**Introduction:**

The assessment of online health‐related information presents a significant challenge for today's youth. Using the Processing of Commercial Media Content (PCMC) model, we investigated the impact of advertising on the perceived credibility of informational content concerning fitness (in boys) and dietary supplements (in girls).

**Methods:**

In a survey‐based preregistered experiment, 681 Czech adolescents (aged 13–18, 52% girls) were randomly assigned to three groups and exposed to websites with a banner ad, a native ad, or simple informational text without an ad.

**Results:**

The presence of ads on websites diminishes the perceived credibility of informational content. While we did not observe the difference between banner and native advertising, we found one gender‐specific difference. Specifically, boys perceive informational content linked with native ads as more credible compared to girls. Additionally, no difference was found between younger and older adolescents. Adolescents demonstrated success in identifying both types of advertisements, irrespective of their age.

**Conclusions:**

The findings are discussed in light of individual differences and various approaches to processing online content. It appears that adolescents are accustomed to native advertising, which seamlessly integrates with traditional banner ads. Moreover, it is plausible that boys assess advertising texts more heuristically and less systematically than girls. Contrary to PCMC expectations, even younger adolescents seem to possess sufficient skills to identify the advertisements.

Currently, the vast majority of adolescents turn to online sources for health‐related information (Sbaffi & Rowley, 2017). While the internet provides easy access to information, it also harbors content that may endanger adolescents' health. One specific concern is advertising content (Lahav & Zimand‐Sheiner, 2016; Lapierre et al., 2017). This particularly applies to advertisements promoting health‐related products like fitness and dietary supplements, which have been linked to an increased risk of serious health issues (Or et al., 2019). These advertisements often lack a solid grounding in research (Grunewald & Bailey, 1993), promote products for older populations (Herriman et al., 2017), and tend to conceal the side effects (Pomeranz et al., 2015). The current media landscape trends toward seamlessly integrating advertising content into one unified context, making it less recognizable and more pertinent (Buijzen et al., 2010). Yet, studies investigating the effects of modern health and fitness advertising on adolescents remain limited (Buijzen et al., 2010). Consequently, there's a pressing need for research on the impact of persuasive advertising on adolescents. Our research aims to enhance understanding of how adolescents evaluate informational content related to fitness and dietary supplements, considering that the publishers' intent might be to sell the product rather than to inform.

A crucial factor of this assessment involves assessing the credibility of online informational content. The perceived credibility is defined as the “believability of some information and/or its source,” with primary dimensions encompassing perceived trustworthiness and expertise (Metzger, 2007, p. 2078). Perceived credibility impacts recipients' behaviors, such as purchase intentions (Flanagin et al., 2014; Jäger & Weber, 2020; Pornpitakpan, 2004), thereby serving as an intermediary of persuasion in advertising. However, due to adolescents' (especially early adolescents') still‐developing knowledge of online advertising persuasion techniques, they often exhibit lower levels of critical thinking (Buijzen et al., 2010; van Dam & van Reijmersdal, 2019; Van Reijmersdal & van Dam, 2020). Consequently, they may be more susceptible to the potential negative influences of advertisements. This worrisome scenario underscores the need for a more comprehensive examination of the factors influencing adolescents' perceptions of informational content within advertising contexts.

To understand how adolescents evaluate the credibility of such informational content, we draw on the Young People's Processing of Commercial Media Content (PCMC) model (Buijzen et al., 2010). This model elucidates how persuasion develops based on the information the audience encounters. Building upon this model and corroborating evidence, we propose that exposure to information and advertising demanding higher cognitive effort (systematic processing) will enhance the perceived credibility of health and fitness‐related content. Conversely, advertising content requiring lower cognitive effort (heuristic processing) will diminish the perceived credibility of such content.

This study specifically investigates the impact of advertising on adolescents' perceived credibility of online informational content pertaining to fitness/dietary supplements. Previous research has shown that the mere presence of ads (even nonrelated) on websites providing information about health products reduced the perceived credibility of offered information (for a review, see Sbaffi & Rowley, 2017). However, these studies typically focused on the effects of inappropriate, visually disturbing, and unrelated pop‐up, interstitial, and banner ads, which are either ignored (i.e., banner blindness; Cho & Cheon, 2004) or trigger more negative responses (Lee & Ahn, 2012; Liu et al., 2018). Therefore, our study not only examines the impact of simple informational text without advertisement (no ad text), but primarily concentrates on two types of modern commercial advertisements: nonintrusive content‐related banner advertisements, and native advertisements. Native advertisements are more persuasive, subtler, less evident, and less noticeable, and they mimic the credibility, form, and appearance of the original sources of the ad publishers (Wojdynski & Evans, 2016).

Drawing from prior research (Sbaffi & Rowley, 2017), we re‐examine the effect of advertisements on adolescents' assessment of the credibility of health‐related information. Our study specifically examines advertisements that are relevant to the topic, visually undisturbed, and particularly modern and appealing. Through this approach, we aim to provide new evidence about the impact of current advertising strategies on perceptions of credibility. In our investigation, we also consider individual factors that may shape perceptions of credibility, such as eHealth literacy and general Trust in Online Health Information (TOHI). Additionally, we explore the potential effects of gender and age and also inquire about purchase intention and advertisement identification.

## 1 YOUNG PEOPLE'S PCMC MODEL AND ADVERTISING CREDIBILITY

The perception of credibility arises from an evaluation process within the context of information persuasion. Advertising represents a deliberate attempt to persuade (Richards & Curran, 2002), aiming to instill a sense of credibility in the presented information or the advertised product, ultimately impacting consumption behavior or brand/product relationships (Hussain et al., 2020). The PCMC model is one of many dual models of information persuasion (Buijzen et al., 2010; Metzger & Flanagin, 2015), positing that information persuasion operates through two pathways: “systematic processing” (involving intricate critical evaluation of information, requiring high motivation and cognitive effort) and “heuristic processing” (relying on simplified clues, with minimal motivation and cognitive effort).^1^ However, these two evaluation processes can operate independently and simultaneously (Chen et al., 1999), and manipulating respondents' motivation levels poses challenges and potential validity issues. Therefore, we do not aim to manipulate motivation levels in this study but rather focus on the nature of the content (Baumeister & Vohs, 2007; Papyrina, 2015).

### 1.1 Complexity and commerciality of content

In the literature, we can delineate two axes along which we can hypothesize the persuasion effect, as indicated by the degree of credibility in the fitness/dietary supplement information and advertising: the complexity of content and its commerciality.

In relation to the complexity of the content, we utilize recent studies on the persuasion of health information (e.g., Ju & Zhang, 2020; Muela‐Molina et al., 2020). We assume that systematic processing is associated with more complex direct product‐related information requiring rational evaluation (in our case, no ad text and native advertising), contrasting with cues that appeal more to quick and superficial evaluation (in our case, banner advertising). In other words, with no ad text and native advertising, we posit that consumers need to engage in a more cognitively demanding process to obtain information and make credibility assessments. Conversely, visible banner ads allow for quicker processing of advertising content with minimal cognitive effort.

The relationship between credibility and type of content (processing method, respectively) is further clarified by evidence of the influence of commerciality, which is therefore the second axis affecting credibility. In this context, commerciality can be understood as the nature of content that primarily functions to generate profit. This characteristic is recognizable, for example, by the nature of the source as a commercial producer or seller, but also by its content focused on a “product” treated as a commodity that can or should be acquired by purchase (cf. Johnson, 1999; Van Reijmersdal & van Dam, 2020). Conventionally, advertising is the major form of commercial content.

Despite rare evidence of a positive effect of advertising on the perceived credibility of content on websites (Fogg et al., 2003), most studies indicate the opposite tendency. Signs of commerce have emerged as primary factors in the rejection of websites by individuals seeking health information (Morahan‐Martin, 2004; Sbaffi & Rowley, 2017). A decline in the perceived credibility and objectivity of health information was also evident in the study by Chang et al. (2021). The presence of advertising diminishes the credibility of health information (or its sources) because it acts as a clue for rapid, cognitively efficient evaluation which can be referred to as a specific type of “commerciality‐penalizing heuristic” for assessing credibility. In this case, the signs of commerciality function as indicators of the side interests associated with the primary informational content. Thus, advertising content tends to trigger fears of manipulation by unknown background actors. In some cases, the activation of this heuristic leads to a complete rejection of the source (e.g., the website; Metzger et al., 2010). Fogg et al. (2003, 2001) reported similar findings, suggesting that the diminished credibility of the informational content is due to the obscuring of the information function of the content by commercialization.

Thus, our theoretical framework suggests that credibility varies based on the degree of cognitive effort required to evaluate the presented content and the extent of its commerciality. In light of these findings, we consider both banner and native advertising as content that elicits heuristic processing because they display visible signs of commerciality that reduce the credibility of the informational content. Conversely, content that includes complex product information without commerciality (no ad text) should trigger systematic processing, resulting in a relatively higher persuasive effect as indicated by increased credibility. Therefore, in hypotheses H1.1 and H2.1, we assume that content subject to systematic processing without commerciality (no ad text) will be perceived as more credible, whereas content subject to heuristic processing with high commerciality (banner and native ads) will be perceived as less credible.

### 1.2 Advertising type and credibility

Nevertheless, the simple logic that equates advertising with heuristic processing and low credibility is complicated by developments in the field of advertising. Actually, the level of content commerciality can vary. The advertising industry is increasingly aware of the negative audience reaction to clearly identifiable and visible advertisements, such as unrelated, pop‐up, interstitial, and banner ads (Lee & Ahn, 2012; Liu et al., 2018). This awareness has led to the promotion of less recognizable and better‐targeted advertising, known as native advertising. The goal of native advertising is to prevent the activation of commerciality penalizing heuristic and the rapid discrediting and avoidance of the content by the audience. The key technique is to decrease perceived commerciality by mimicking the credibility, form, and appearance of original sources of publishers (Boerman et al., 2014; Campbell & Evans, 2018; Harms et al., 2017, 2019; Wojdynski, 2016; Wojdynski & Evans, 2016; Wojdynski & Golan, 2016). While the decline in credibility is well‐documented (Dubowicz & Schulz, 2015; Eysenbach, 2002; Sillence et al., 2007), the impact of native advertising on credibility remains less clear.

The fact that native ads are often hidden and their purpose undisclosed makes them harder to recognize, which has theoretical implications. In relation to the PCMC model of persuasion, native advertising represents a “liminal case” between systematic and heuristic processing, thus affecting perceived credibility. Due to its high level of integration of advertising into the context, native advertising poses particular challenges for adolescents' recognition abilities (Buijzen et al., 2010). This allows for systematic processing based on attentive engagement with the relatively complex text without noticing the embedded advertisement. However, efforts to reduce the level of commerciality may not always be successful. Confrontation with native advertising can still activate heuristic processing that penalizes commerciality.

Our study, therefore, aims to investigate the impact of different types of advertising content (banner and native) on their perceived credibility. However, since there is no solid evidence to formulate a clear assumption about the impact of native advertising (compared to no ad text and banner advertising) on credibility, our hypotheses are exploratory. Hence, in hypotheses H1.2 and H2.2, we assume that the perceived credibility of informational content varies based on the presence of banner and native advertising.

### 1.3 Individual factors of perceived credibility

In addition to the mentioned hypotheses, we considered several individual factors that could potentially influence perceived credibility. Although evidence of gender differences in the perception of web advertising is sparse, existing differences appear to be statistically insignificant (Berry & Shields, 2014) or at least practically negligible (see Fogg et al., 2001 2). Therefore, in hypotheses H1.3 and H2.3, we assume a lack of practically and statistically significant differences between boys and girls in their perceptions of credibility.

Furthermore, as indicated by the PCMC model and other evidence, adolescents' ability to recognize hidden advertising is not fully developed (van Dam & van Reijmersdal, 2019; Van Reijmersdal & van Dam, 2020). Therefore, in hypothesis H3, we assume that older participants are more likely to identify the presence of advertisements compared to younger participants. This assumption is based on the idea that older individuals have greater experience and capacity to process content systematically, making them more resistant to cognitive overload and less prone to heuristic processing when encountering more complex content.

## 2 METHODS

### 2.1 Plan of analysis

We pre‐registered three main hypotheses and six elaborating hypotheses, along with the related statistical procedures (see https://osf.io/4pvz6/). Any deviations from the preregistered plan are documented in Supporting Information S1: Data A. For the analysis of the first and second hypotheses, we utilized multivariate analysis of covariance (MANCOVA) to detect the difference between groups in credibility subscales on a multivariate level, followed by two subsequent analysis of covariances (ANCOVAs) to detect the differences on a univariate level. Planned contrasts were then employed to detect the specific pairwise differences between groups in both credibility subscales, with the Holm–Bonferroni correction applied to reduce type I errors. To test the third hypothesis, Fisher's exact test was utilized. All analyses were performed in *R* (v4.0.3; R Core Team, 2020) using packages *rstatix* (Kassambra, 2021), *MASS* (Venables & Ripley, 2002), *assertr* (Fischetti, 2020), *effectsize* (Ben‐Shachar et al., 2020), and *emmeans* (Lenth, 2020). The data, materials, questionnaire, translations, and the *R* syntax are available online (https://osf.io/khqat/).

### 2.2 Experimental stimuli

In relation to health and body‐related advertising, previous studies (Berry & Shields, 2014; Grogan, 2017) have demonstrated that advertising communication strategies are gendered differently. Men and women are exposed to distinct representations regarding the body parts and aspirations they are encouraged to modify (Dworkin & Wachs, 2009). Conversely, men and women dissatisfied with the state of their bodies tend to have different aspirations—men primarily focus on enhancing upper body musculature and prioritize increasing body mass, whereas women predominantly seek to shape the lower half of the body and prioritize achieving slimness (Grogan, 2017).

**Figure 1:**
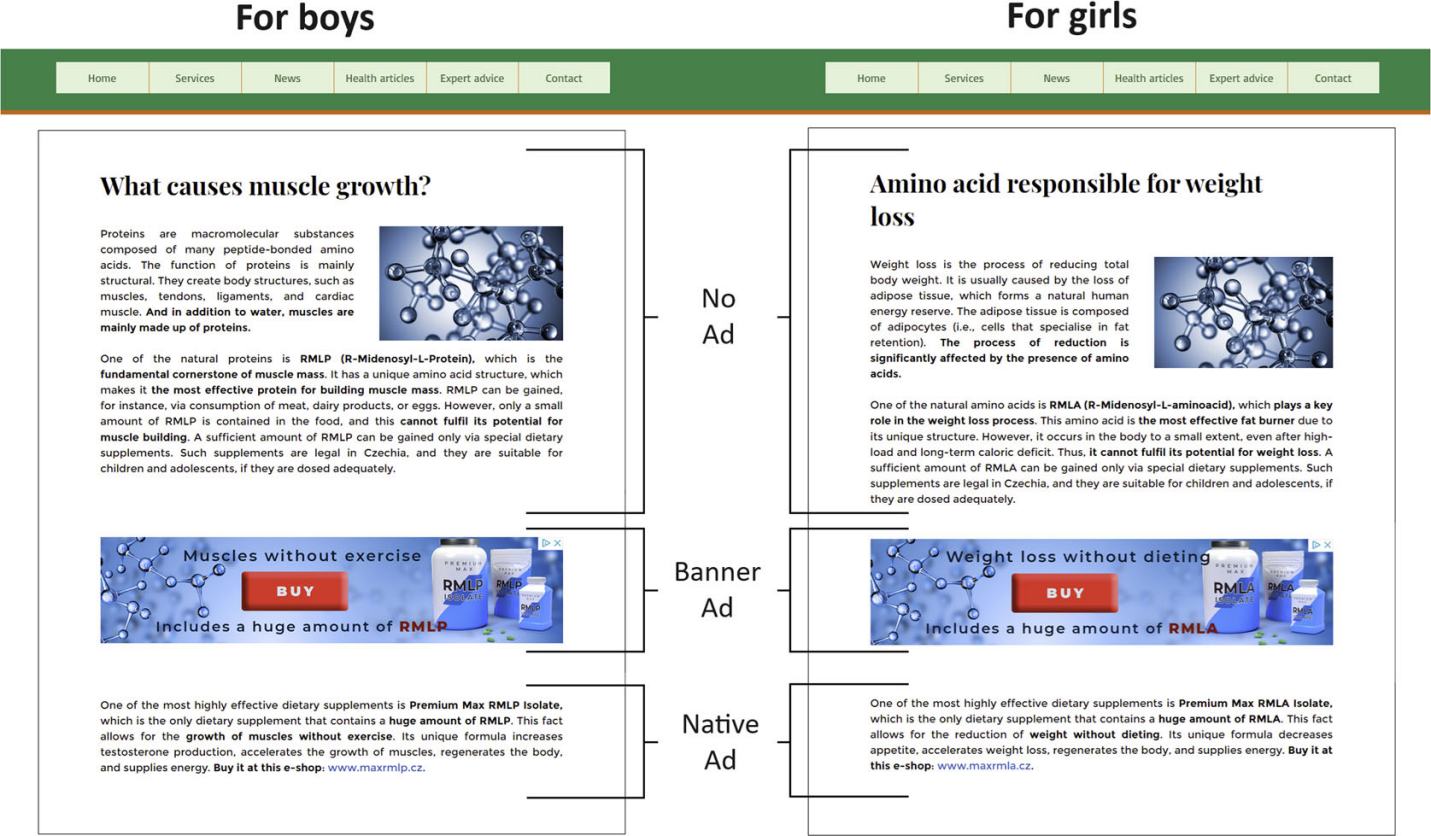
Experimental stimuli.

Hence, to enhance the internal validity, we developed three different gendered stimuli displayed on a fictional website focused on fitness and a healthy lifestyle (Figure 1). The text pertained to fitness/dietary supplements, featuring a fictitious active substance purportedly enhancing muscle‐building (for boys) and weight‐loss (for girls). To ensure balanced content, we included accurate information alongside a few fictional ones. Specifically, participants were introduced to fictional information about an active substance called “R‐Midenosyl‐l‐Protein/R‐Midenosyl‐l‐Aminoacid.” Its crucial role in muscle growth/fat burning was emphasized, and it was explained that the substance could only be obtained in negligible amounts naturally. As a result, it was mentioned that supplements were necessary to fully utilize its potential. This approach was identical for all conditions and was essential to mitigate the potential impact of diminished credibility stemming from unrealistic promises presented in banner and native advertising, rather than solely from the presence of advertising itself.

The “no ad text group” was exposed to a stimulus comprising informational text devoid of specific product information or promotion. The remaining two stimuli contained commercial elements. The second stimulus included an additional banner ad for a particular fitness/dietary supplement, clearly delineated from the textual content (i.e., “banner ad group”). The third stimulus expanded the text by referencing specific products (with a hyperlink to an e‐shop) without explicit separation and devoid of any indication of the sponsored content (i.e., “native ad group”). The website background was anonymous, but was designed to resemble other health‐related websites (e.g., green header).

## 3 MEASURES

### 3.1 Perceived credibility of information

The dependent variables were measured by two subscales of TOHI (Johnson, Rowley, et al., 2015; Johnson, Sbaffi, et al., 2015; Rowley et al., 2015), namely the credibility subscale consisting of five items and the reliability of the content subscale consisting of four items. The credibility subscale measures subjectively perceived believability, quality, and the morality of the information (e.g., “Whether I feel I can believe the information.”), while the reliability of the content subscale measures the core characteristics of the information such as accuracy, comprehensiveness, and objectivity (e.g., “The reliability of the information.”). These two subscales correspond to the traditional dimensions of trustworthiness and expertise (Metzger, 2007). Participants answered on a 5‐point Likert scale ranging from 1 (*Strongly disagree*) to 5 (*Strongly agree*). Both subscales showed satisfactory reliability (credibility subscale: ω = 0.794; reliability content subscale: ω = 0.723).

### 3.2 Control variables

*eHealth Literacy Scale* (eHEALS; Norman & Skinner, 2006): We used six items from the scale to measure health literacy (e.g., “I can tell high quality health resources from low quality health resources on the internet.”) on a 5‐point scale (1 = *Strongly disagree*, 5 = *Strongly agree*). Its internal consistency was satisfactory (ω = 0.828).

*Trust in Health Information Websites Scale* (THIW; Sheng & Simpson, 2015): Five items were employed to measure the general trust in online health websites (e.g., “My overall faith in health websites is high.”) on a 5‐point scale (1 = *Completely untrue*, 5 = *Completely true*). The scale demonstrated very high internal consistency (ω = 0.910).

Newly developed *eHealth Sources Online Seeking* (eHSOS) scale: Six items were used to measure the frequency of seeking health information (e.g., “Dietary supplements or vitamins”) measured on a 6‐point scale (1 = *Never*, 6 = *Several times a day*). Its reliability was satisfactory (ω = 0.843).

Newly developed *Health Online Shopping* (HOS): Four items measured the frequency of purchasing health products online (e.g., “Weight loss products”) on a 4‐point scale (1 = *Never*, 4 = *Several times a week*). Its reliability was also sufficient (ω = 0.833).

Additionally, data were collected on advertising disclosure (e.g., “Did the website contain advertising?”), substance purchase intention (e.g., “Would you buy a supplement containing a high amount of [name of active substance]”), and product purchase intention (e.g., “Would you buy the [name of product]?”). Information regarding the gender and the age of the participants was also recorded.

### 3.3 Sample and procedure

This study was conducted as part of a larger investigation into digital technologies. Data collection took place online in November 2020, involving 1530 participants from the Czech Republic. Participants were recruited through an established online panel managed by a research agency. Nonprobabilistic quota sampling was utilized to ensure balanced proportions of gender, age, age versus gender combinations, household socioeconomic status (measured as family income), and place of residence (NUTS3). Each household received approximately 4€ for participation. For this study, approximately half of the participants (*N* = 872) were randomly selected, while the rest were part of a different experimental study. The study received approval from the Research Ethics Committee of the Masaryk University. Informed consent was obtained from both parents and participants by the research agency.

We conducted a between‐subjects experiment with three experimental conditions. Participants were randomly assigned (separately for both genders) to one of the three equally sized predetermined blocks using block randomization. All participants were exposed to experimental stimuli for at least 20 s (*median* = 49.9 s, interquartile range [IQR] = 45 s), after which they provided data on selected measures.

From the original subsample, we excluded 168 participants due to a high number of missing values (i.e., more than three missing values in each credibility subscale); 10 participants were identified as multivariate outliers using Mahalanobis distance; and an additional 13 participants were identified as univariate outliers (i.e., values beyond Q1 or Q3±1.5×IQR). Consequently, data from 681 participants were analyzed. This sample size provides sufficient statistical power as indicated by a priori power analysis (see Supporting Information S1: Data B).

The analyzed sample was categorized as follows: age range 13–18 (*M* = 15.3, *SD *= 1.7); 52% girls; 41.6% students of elementary school (*N* = 283); 33.3% secondary vocational school (*N* = 227); 22.8% grammar school (*N* = 155); and 2.3% who were at different levels of education or were not students (*N* = 15).

## 4 RESULTS

In the first step, we assessed the psychometric properties of the utilized scales, focusing on their factor structure and internal consistency (see Supporting Information S1: Data C). Subsequently, we examined the assumptions of MANCOVA with three experimental conditions as independent variables, two TOHI subscales as dependent variables, and age, and eHEALS, eHSOS, HOS, and THIW as covariates. Although some assumptions of MANCOVA were slightly violated (see Supporting Information S1: Data D), we addressed this by: (1) utilizing robust Pillai's Trace MANCOVA statistics; (2) excluding three covariates (i.e., eHEALS, eHSOS, and HOS) from the model; (3) employing Box‐Cox transformation for ANCOVA statistics; and (4) validating ANCOVA results through a robustness check using nonparametric ANCOVA (Young & Bowman, 1995; see Supporting Information S1: Data E).

H1 and H2 The differences between experimental conditions are both statistically and practically significant (*p* < .05 and ES > 0.20) for both TOHI subscales.

MANCOVA indicated a statistically and practically significant difference between the experimental conditions (i.e., no ad text, banner ad, and native ad) on the dependent variables (TOHI subscales); Pillai's trace = 0.036, *F*(4, 1348) = 6.094, *p* < .001, ω^2^ = 0.02, after controlling the influence of THIW [Pillai's trace = 0.044, *F*(2, 673) = 15.581, *p* < .001, ω^2^ = 0.04] and age [Pillai's trace = 0.005, *F*(2, 673) = 1.572, *p* = .208, ω^2^ < 0.01].

Two subsequent ANCOVAs were performed with Box‐Cox transformation (credibility λ = 1.1919; reliable content λ = 0.9899). The differences between the experimental conditions were statistically significant and demonstrated large effect sizes. For the credibility subscale, *F*(2, 674) = 8.946, *p* < .001, ω^2^ = 0.02, while controlling for the influence of THIW [*F*(1, 674) = 30.474, *p* < .001, ω^2^ = 0.04] and age [*F*(1, 674) = 1.148, *p* = .284, ω^2^ < 0.01]. For reliable content, *F*(2, 674) = 11.940, *p* < .001, ω^2^ = 0.03, while controlling for the influence of THIW [*F*(1, 674) = 18.014, *p* < .001, ω^2^ = 0.02] and age [*F*(1, 674) = 3.134, *p* = .077, ω^2^ < 0.01]. The robustness check within nonparametric ANCOVAs also showed statistically significant results (see Supporting Information S1: Data E). Based on these findings, we can conclude that H1 and H2 were supported.

H1.1 and H2.1 Groups without ads show statistically higher credibility and reliable‐information assessment than groups with ads.

Upon ANCOVAs, statistically significant results were obtained, and the post‐hoc tests were calculated on the estimated marginal means (i.e., EMM) while controlling for THIW and age. The group without ads showed higher credibility (credibility: EMM = 3.53, *SE* = 0.045; reliable content: EMM = 3.14, *SE* = 0.044) compared to groups with banner (credibility: EMM = 3.31, *SE* = 0.046; reliable content: EMM = 2.88, *SE* = 0.045) and native ads (credibility: EMM = 3.29, *SE* = 0.046; reliable content: EMM = 2.87, *SE* = 0.045). These differences were statistically significant; for the difference between no ad text and banner ad (credibility: *p*_Holm_ = 0.001, *d* = 0.321; reliable content: *p*_Holm_ < 0.001, *d* = 0.384) and for the difference between no ad text and native ad (credibility: *p*_Holm_ < 0.001, *d* = 0.354; reliable content: *p*_Holm_ < 0.001, *d* = 0.402). Although these effect sizes were relatively small, they still indicate the practical significance of the results according to the pre‐registered threshold, thus supporting H1.1 and H2.1.

H1.2 and H2.2 Groups with banner and native ads statistically differ in their level of credibility and reliable information assessment.

*N*o significant difference was found for the credibility subscale and the reliable‐content assessment (credibility: *p*_Holm_ = 0.724, *d* = 0.033; reliable content: *p*_Holm_ = 0.849, *d* = 0.018). Both native and banner ads elicited similar assessments of the credibility among participants (*EMMs* are provided in the previous section). Therefore, H1.2 and H2.2 were not supported.

H1.3 and H2.3 Differences between boys and girls across all groups in the level of credibility and reliable‐information assessment are statistically and practically insignificant.

For the purposes of examining the gender differences, additional MANCOVA and two separate ANCOVAs with six instead of three experimental conditions were performed, aiming to clearly distinguish between gender‐specific conditions (i.e., no ad text boys, no ad text girls, banner ad boys, banner ad girls, native ad boys, and native ad girls). The results were nearly identical to those presented in the main analysis (H1 and H2 section).

MANCOVA revealed a statistically and practically significant difference between the experimental conditions on the combined dependent variables (TOHI subscales); Pillai's trace = 0.073, *F*(10, 1342) = 5.064, *p* < .001, ω^2^ = 0.03, after controlling the influence of THIW [Pillai's trace = 0.038, *F*(2, 670) = 13.250, *p* < .001, ω^2^ = 0.04] and age [Pillai's trace = 0.005, *F*(2, 670) = 1.584, *p* = .206, ω^2^ < 0.01]. Two subsequent ANCOVAs with Box–Cox transformation (credibility λ = 1.1919; reliable content λ = 0.9494) also showed statistically significant differences between six experimental conditions and these differences had large effect sizes. For the credibility subscale, *F*(5, 671) = 5.766, *p* < .001, ω^2^ = 0.03, while controlling for the influence of THIW [*F*(1, 671) = 26.136, *p* < .001, ω^2^ = 0.04] and age [*F*(1, 671) = 1.146, *p* = .285, ω^2^ < 0.01]. For reliable content, *F*(5, 671) = 10.720, *p* < .001, ω^2^ = 0.07, while controlling for the influence of THIW [*F*(1, 671) = 12.854, *p* < .001, ω^2^ = 0.02] and age [*F*(1, 671) = 3.193, *p* = .074, ω^2^ < 0.01].

**Figure 2:**
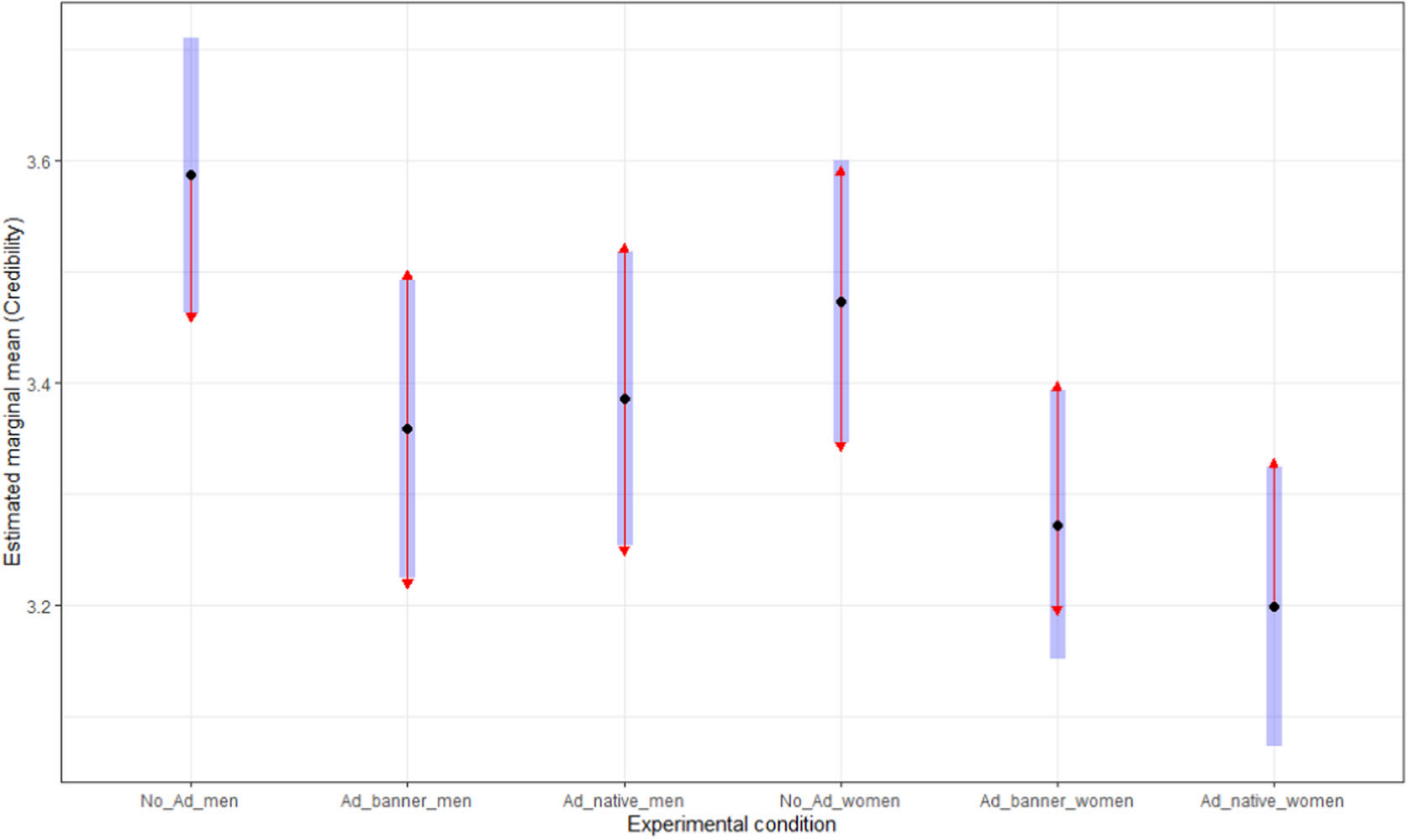
Planned contrasts for the credibility subscale.

**Figure 3:**
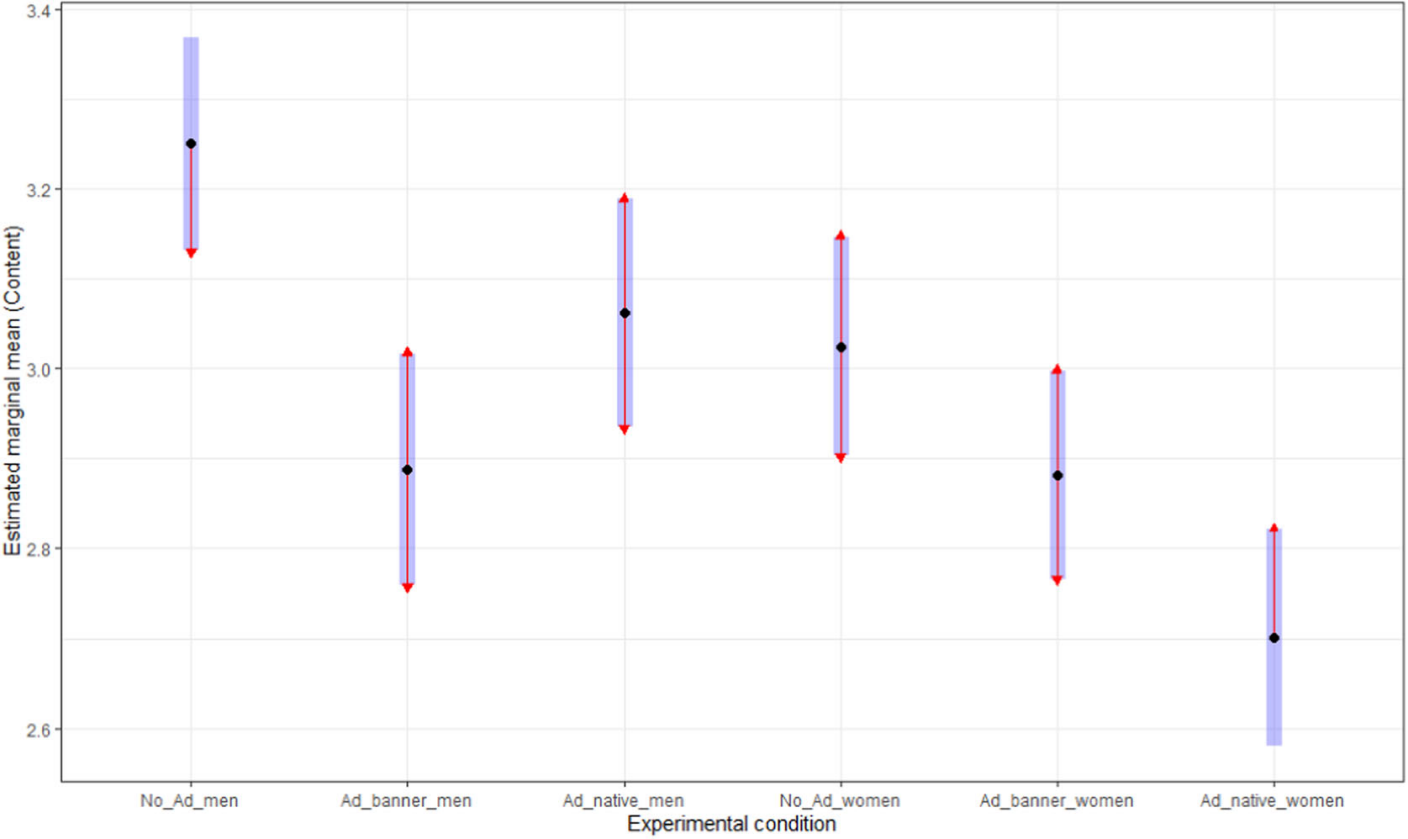
Planned contrasts for the reliable‐content subscale.

Hence, we proceeded with planned contrast pairwise comparisons (see Table 1 and Figures 2 and 3). We found no significant differences in the credibility subscale between boys and girls for all experimental conditions. Hence, the H1.3 was supported. Regarding the reliable‐content subscale, statistically significant differences between boys and girls were identified in the native ad (*p*_Holm_ < 0.001, *d* = 0.549) and in no ad text (*p*_Holm_ = 0.057, *d* = 0.342) conditions. In both cases, boys (no ad text: EMM = 3.250, *SE *= 0.060; native ad: EMM = 3.062, *SE *= 0.064) assessed the stimuli as more reliable than girls (no ad text: EMM = 3.025, *SE *= 0.062; native ad: EMM = 2.700, *SE *= 0.061). Therefore, we consider that H2.3 was not supported.

**Table 1:** Planned contrasts for gender differences.

Scale	Boys vs. girls	Difference between EMM [95% *CI*]	*SE*	*p*_Holm_	*d* [95% *CI*]
Credibility subscale	No ad text	0.114 [−0.064, 0.291]	0.090	0.417	0.166 [−0.093, 0.424]
	Banner ad	0.086 [−0.094, 0.267]	0.092	0.417	0.126 [−0.138, 0.389]
	Native ad	0.187 [.004, 0.370]	0.093	0.135	0.273 [0.006, 0.540]
Reliable content subscale	No ad text	0.224 [−0.055, 0.395]	0.086	0.019	0.342 [0.083, 0.601]
	Banner ad	0.006 [−0.167, 0.179]	0.088	0.946	0.009 [−0.254, 0.272]
	Native ad	0.361 [0.186, 0.537]	0.089	<0.001	0.549 [0.281, 0.818]

Abbrevaitions: *CI*, confidence intervals; *d*, Cohen's *d*; EMM, estimated marginal means; *p*_Holm_, *p* value with Holm–Bonferroni correction; *SE*, standard error.

Older participants statistically more often identify the presence of advertisements than younger participants.

We performed Fisher's exact test with 10,000 bootstraps to verify H3. The comparison between 13–15‐ and 16–18‐year‐olds showed no significant differences (*p* = .569, odds ratio [*OR*] = 0.895 [95% confidence interval [*CI*]: 0.603, 1.323]). This also applied for separate analyses within the groups with the banner ad (*p* = .232, *OR* = 0.387 [95% *CI*: 0.065, 1.611]) and the native ad (*p* = 1.000, *OR *= 0.949 [95% *CI*: 0.399, 2.208]). Hence, H3 was not supported.

### 4.1 Descriptive statistics of intention to buy and ad disclosure

As is evident from Table 2, the vast majority of the participants identified banner advertising, with only slightly fewer identifying native advertising. Concerning purchase intention, a similar pattern emerged in responses, which aligns with the findings of H2.3: boys demonstrated higher intentions to buy products advertised within the native format compared to girls.

**Table 2:** Descriptive statistics.

Group	Substance purchase intention	Product purchase intention	Ad disclosure
No ad text boys	29 (27.9%)		34 (36.2%)
No ad text girls	22 (24.7%)		42 (46.7%)
Banner ad boys	15 (17.2%)	9 (10.5%)	87 (94.6%)
Banner ad girls	11 (10.4%)	10 (9.3%)	107 (93.9%)
Native ad boys	31 (35.2%)	18 (20.7%)	74 (82.2%)
Native ad girls	15 (15.8%)	13 (13.4%)	88 (85.4%)

*Note*: Substance purchase intention—The percentage and number of participants within each group who reported an intention to purchase substances as described in the text. Product purchase intention—The percentage and number of participants within each group who reported an intention to purchase advertised products. Ad disclosure—The percentage and number of participants within each group who recognized the presented content as an ad.

## 5 DISCUSSION

In this pre‐registered study, we examined the impact of advertisements on adolescents' perceptions of the credibility of online health informational content. Specifically, we focus on understanding the influence of both banner and native ads on the perceived credibility and reliability of fitness‐oriented content for boys and diet‐oriented content for girls. Our findings indicate that the presence of banner and native ads, which evoke heuristic processing, diminishes the perception of credibility compared to content without ads. These results are consistent with the PCMC model, which posits a relationship between cognitive engagement levels and cues of commerciality (Buijzen et al., 2010). Notably, heuristic cues, indicative of commerciality tend to undermine the credibility of the content, as suggested by prior research (Morahan‐Martin, 2004; Sbaffi & Rowley, 2017).

However, we observed no significant differences in the credibility of content with a banner compared to content featuring native advertising. Despite the aim of native advertising to seamlessly blend with surrounding text and potentially mitigate commerciality‐penalizing heuristic processing (Boerman et al., 2014; Campbell & Evans, 2018; Harms et al., 2017, 2019; Wojdynski, 2016; Wojdynski & Evans, 2016; Wojdynski & Golan, 2016), our results suggest that it is comparably “unsuccessful” in terms of credibility when compared to text without advertising. In essence, for adolescents, this form of advertising appears to functionally blend in with traditional banner advertising. This observation is reinforced by the comparable detection levels of both types of ads by adolescents. We speculate that the adolescents' familiarity with native advertising contributes to their ability to quickly recognize its nature, akin to banner advertising, thus resulting in a similar perception of credibility.

This finding partially aligns with the results of Johnson et al. (2019), who found that peer‐generated advertising was perceived as the most credible, followed by native advertising, with traditional advertisements rated as the least credible. Therefore, it appears that the online environment may provide a degree of protection against the effects of advertising on adolescents, as it does not permit the involvement of an even more influential marketing type—social advertising—which introduces a new element into heuristic evaluation (the familiar person clue). Consequently, our findings may support the need for increased regulation of advertising on social networking sites compared to the broader web.

These findings undergo modification when considering the social comparison effect. In the same study by Johnson et al. (2019), individuals who are inclined to compare themselves to others, show varying effectiveness of advertisement regarding brand attitudes Peer‐generated advertising is found to be the most effective, followed by native advertising, with conventional advertising being the least effective. Hence, future research should investigate whether social comparison operates similarly on the web, which remains a prominent platform where adolescents encounter advertisements.

We also observed that the above‐mentioned pattern regarding credibility assessment varies slightly for boys and girls. Contrary to expectations, boys rated native advertisements as more reliable than girls did on the reliable‐content subscale. Additionally, boys showed higher general (substance) purchase intention as well as specific (product Premium Max RMLP Isolate) purchase intention compared to girls (35.2% vs. 15.8%, and 20.7% vs. 13.4%, respectively). One potential explanation of these gender‐specific findings may lie in evidence suggesting that the native advertising style negatively affects girls' assessment due to heightened perceptions of potential deception in communication, to which women tend to respond more negatively than men (Levine et al., 1992).

Another explanation stems from studies that highlight gendered predispositions to certain types of advertising content processing (e.g., Darley & Smith, 1995; Machackova & Smahel, 2018; Papyrina, 2015). While women tend to approach texts more systematically, assessing a broader range of aspects more extensively, men tend to evaluate content more heuristically, focusing on a narrower range of aspects with less effort, relying on the recognition of “thresholds” that subsequently influence their reaction or evaluation of the processed content. Consequently, it is plausible that boys may be less critical of the content presented in the text without advertising and native advertising.

Other studies also suggest that adolescents' purchase intentions are influenced by their ability to skeptically evaluate advertising, closely linked to their internet literacy. Both internet literacy and skepticism about advertising are primarily acquired by adolescents through parental mediation (Vijayalakshmi et al., 2020). However, this mediation occurs differently for girls and boys, with boys experiencing more restrictive mediation compared to girls, typically involving limitations on access and time (Clark, 2011; Lee, 2013). While restrictive mediation is statistically associated with lower exposure to various risks, it also limits the opportunity for developing competence in judging online content (Steinfeld, 2021).

Given that prior experience with native advertising is a pivotal factor in the success of persuasive functions (Jung & Heo, 2019), it is plausible that boys may be disadvantaged in their ability to discern the persuasive function of advertisement. Consequently, the gender difference observed in the heuristic‐systematic processing of advertising content and its manifestation in boys' greater willingness to buy may stem from learned differences in the ability to judge online advertising. Thus, confirming the effect of gendered parental mediation on descendants' ability to discern the persuasive function of advertisement warrants further research. If this mechanism is confirmed, it is worth considering incorporating this knowledge into the strategies of campaigns targeting the mediating role of parents and schools. The ultimate goal of such campaigns should be to reduce the gender gap in the mediation treatment between girls and boys in the online space concerning the addressed threats and opportunities (see, e.g., Steinfeld, 2022).

Finally, the assumption regarding the weaker ability of younger adolescents to detect advertising was not supported. Our findings indicate that participants were able to identify advertisements with high success rates, even in the case of no ad text. This ability was consistent across gender and age, suggesting that even younger adolescents likely possess sufficient skills to identify the advertisements (cf. van Dam & van Reijmersdal, 2019; Van Reijmersdal & van Dam, 2020).

One concern raised by the PCMC model, considering the developmental characteristics of adolescents, was that the trend toward high contextual integration of native advertising might lead to increased persuasion, especially among younger adolescents (Buijzen et al., 2010). However, our study did not confirm this concern. Since 12‐year‐old adolescents likely already possess the necessary skills for recognizing advertisements and their persuasive intents (Carter et al., 2011), the lack of difference between younger (13–15 years) and older (16–18 years) adolescents in our study is understandable. However, the mere recognition of native advertisement may not suffice, as disclosure did not automatically elicit persuasion knowledge and a subsequent critical view of the product (Balaban et al., 2022; Eisend et al., 2019).

## 6 LIMITATIONS AND FUTURE DIRECTIONS

Nevertheless, it's important to contextualize our findings within the study's limitations. First, some potentially relevant variables, such as the individual importance of presented ads and products, were not included in this study. These could be explored in future studies as individual factors contributing to a deeper understanding of assessment differences.

Second, the methodological design used might also decrease the ecological validity. Specifically, the assessment conditions within a study context may differ from those in the daily online behavior of youth. Additionally, the presented website was anonymous and did not resemble any well‐known website or influencer, diverging from the youths' common experiences. This restricts the potential effect of native ads that leverage credibility from the sources already perceived as credible. The real influence of native ads presented on well‐known websites might, therefore, be more persuasive and far‐reaching than observed in this study.

Particularly, PCMC hypothesizes that the influence of peer groups and other influential individuals is significant for persuasion success, especially among older adolescents. Thus, the involvement of the mediation aspect (e.g., on social network sites) may yield additional valuable results. Furthermore, the aforementioned theoretical model also assumes a greater involvement of situational and content context, which influences advertising processing (e.g., through perceived integration degree, and remaining cognitive capacity). Including similar stimuli in different contexts (e.g., measures of experienced health/fitness, measures of the ad's entertainment value) can provide not only substantive but also theoretical insights.

## AUTHOR CONTRIBUTIONS

**David Lacko**: Conceptualization; methodology; formal analysis; writing—original draft. **Hana Machackova**: Conceptualization; methodology; writing—review and editing; supervision; funding acquisition. **Lukáš Slavík**: Conceptualization; writing—original draft; writing—review and editing.

## CONFLICT OF INTEREST STATEMENT

The authors declare no conflict of interest.

## ETHICS STATEMENT

The study has been approved by the Masaryk University Ethical Board.

## Supporting information

Supporting information.

## Data Availability

The data that support the findings of this study are openly available in Open Science Framework at https://osf.io/khqat/.

## Footnotes

1 We intentionally leave out consideration of automated processing that works unconsciously due to the difficulty in identifying it.

2 Even though the authors do report neither effect size nor necessary information about group variances to calculate the effect size, based on the used 6‐point scale, reported sample size of 1440 participants and average mean difference of 0.11, we consider the significant results as practically insignificant.
