# Supplementary material for: Adolescents' perceptions of the credibility of informational content on fitness and dietary supplements: The impact of banner and native advertising
Source: J Adolesc. 2024 Aug 20;96(8):1956–68. doi: 10.1002/jad.12394 (PMC11618704; doi:10.1002/jad.12394)
Supplement: Supplementary file 1 — Supporting information. [file JAD-96-1956-s001.docx]

**Supplementary material A: Deviation from the Pre-Registration**

| Category | Pre-registered | Conducted | Justification |
| --- | --- | --- | --- |
| H1, H2, H1.2 & H2.2 | We pre-registered to verify these hypotheses across all six experimental groups (i.e., no ad boys, no ad girls, banner ad boys, banner ad girls, native ad boys, and native ad girls). | We analysed the differences between three experimental conditions (i.e., no ad, native ad, banner ad) without gender distinction. | We did this because we believe that it is clearer for readers and simultaneously corresponds to the defined hypothesis, despite the fact that we did not specify it in the pre-registration. All six groups were used for testing H1.3 and H2.3. |
| H1.3 & H2.3 | “Difference is practically insignificant.” | We added the wording “statistically significant” to the hypotheses. | We planned to interpret statistical significance as the first step via *p*-values, and then practical significance via effect sizes. |
| Age categories | Not specified. | We created two age categories (13-15 and 16-18). | We neglected to specify the age categories in pre-registration. |
| Fisher’s exact test | Chi-square test or logistic regression. | We used Fisher’s exact test. | We used the Fisher test because it is more suitable for a relatively smaller number of observations per cell. |
| Participant rewards | Approx. 10-20 EUR. | Approx. 4 EUR. | The external agency specified the final price after the pre-registration was conducted. |
| Box-Cox transformation | Non-parametric and robust equivalents of (M)ANCOVA. We planned to use robust ANCOVA in R package “WRS2”. | We used Box-Cox transformation for ANCOVA statistics. In addition, we performed a robustness check with parametric ANCOVA without Box-Cox transformation and with non-parametric ANCOVA. | Unfortunately, robust ANCOVA in WRS2 is currently available only for two groups and non-parametric ANCOVA, which was used as a robustness check, provides only p-values (it is currently not possible to compute effect sizes). Hence, we decided to use Box-Cox transformation within traditional parametric ANCOVA. |
| Univariate outliers | Not specified. | We removed 13 participants as univariate outliers (i.e., values above Q3+1.5*IQR or below Q1-1.5*IQR) from the analysis. | We took this step due to some violations of MANCOVA assumptions (see Appendix D). |
| Pillai’s Trace | Not specified. | We used Pillai’s Trace estimator in MANCOVA. | We used Pillai’s Trace because the homogeneity of variances was slightly violated. |
| MLR | Not specified. | We used MLR estimator in CFA and MG-CFA. | We used MLR because the data showed multivariate non-normality. |
| Covariates | HOS, eHSOS, eHEALS, Age, and THIW. | HOS, eHSOS, and eHEALS were not included. | We removed these covariates from the analysis because their inclusion would violate the MANCOVA assumptions (see Appendix D). |

**Supplementary material B: Power analysis**

A priori power analysis for ANCOVA in *G*Power* (v 3.1.9.7; Faul et al., 2007) was performed. Setting α at .05, power (1-β) at .80, effect size *f* at .20, numerator *df* at 5, number of groups at 6, and number of covariates at 5 was sufficient to test at least 327 participants (55 per group; denominator *df* = 314, critical *F* = 2.243, noncentrality parameter λ = 13.08).

The effect size *f* was chosen on the basis of previous similar studies, which used self-report scales to access website credibility and which somehow manipulated with the presence or form of website advertising. Even though the vast majority of previous research was rather qualitative or descriptive, we were able to extract effect sizes from a few studies and transform them into Cohen’s *f* effect size (the most of effect sizes were computed from reported degrees of freedom and *t* or *F* statistics). These studies found rather weak to moderate effect sizes, the certain *f* values were .490 (Stoeger, 2007), .356 (Zimand-Sheiner et al., 2020), .328 (Dubowicz & Schulz, 2015) .252, .229 (Walther et al., 2004) and 0.201 (Howe & Teufel, 2014). Hence, we choose the lowest found *f* value of .20 (which is a value between weak and moderate effect size; Cohen 1992) as the effect size used in the power analysis.

Faul, F., Erdfelder, E., Lang, A.-G., & Buchner, A. (2007). G*Power 3: A flexible statistical power analysis program for the social, behavioral, and biomedical sciences. *Behavior Research Methods, 39*, 175-191.

Zimand-Sheiner, D., Ryan, T., Kip, S. M., & Lahav, T. (2020). Native advertising credibility perceptions and ethical attitudes: An exploratory study among adolescents in the United States, Turkey and Israel. *Journal of Business Research, 116*, 608–619. <https://doi.org/10.1016/j.jbusres.2019.06.020>.

Dubowicz A., & Schulz, P. J. (2015). Medical Information on the Internet: A Tool for Measuring Consumer Perception of Quality Aspects. *Interactive Journal of Medical Research, 4*(1), e8.

Walther, J. B., Wang, Z., & Loh, T. (2004). The Effect of Top-Level Domains and Advertisements on Health Web Site Credibility. *Journal of Medical Internet Research*, 6(3), e24.

Howe, P., & Teufel, B. (2014). Native advertising and digital natives: The effects of age and advertisement format on news website credibility judgments. *ISOJ Journal, 4*(1), 78-90.

Cohen, J. (1992). A power primer. *Psychological Bulletin*, 112(1), 155–159. <https://doi.org/10.1037/0033-2909.112.1.155>.

**Supplementary material C: Psychometric properties of used scales**

In the first step, a multi-group confirmatory factor analysis and measurement invariance of the TOHI scale were verified. Since the Henze-Zirkler test (*HZ* = 2.995, *p* < .001) as well as the multivariate Shapiro-Wilk test (*SW* = 0.971, *p* < .001) suggested non-normally distributed data, the maximum likelihood estimator with robust standard errors (MLR) and with Full Info Max Likelihood (FIML) procedure to handle missing values were used. Since the fit indices of the proposed models are satisfactory, the factor validity of the TOHI scale is sufficient. Furthermore, measurement invariance across three experimental groups yielded a satisfactory scalar level meaning the comparison of means is possible.

| Level | *RMSEA* [90% *CI*] | *SRMR* | *CFI* | *TLI* | ΔRMSEA | ΔSRMR | ΔCFI | ΔTLI |
| --- | --- | --- | --- | --- | --- | --- | --- | --- |
| Configural (CFA) | .064 [.052, .077] | .031 | .972 | .960 | – | – | – | – |
| Configural (MG-CFA) | .060 [.043, .077] | .035 | .978 | .968 | – | – | – | – |
| Metric (MG-CFA) | .056 [.040, .072] | .051 | .977 | .972 | -.004 | .016 | .001 | .004 |
| Scalar (MG-CFA) | .063 [.048, .077] | .059 | .967 | .965 | .007 | .008 | .010 | .007 |

*note: RMSEA* = root mean square error of approximation, *SRMR* = standardized root mean square residual, *CFI* = Comparative Fit Index, *TLI* = Tucker-Lewis Index; *CI* = confidence intervals, Δ (delta) = change.

In the second step, the internal consistency of all scales was verified. All scales showed sufficient internal consistency (TOHI credibility subscale: ω = .794; TOHI reliability content subscale: ω = .723; HOS: ω = .833; eHSOS: ω = .843; eHEALS: ω = .828; THIW: ω = .910). We used packages *lavaan* (Rosseel, 2012), *semTools* (Jorgensen et al., 2021) and *MVN* (Korkmaz et al., 2014).

Rosseel, Y. (2012). lavaan: An R Package for Structural Equation Modeling. *Journal of Statistical Software, 48*(2), 1–36. <https://www.jstatsoft.org/v48/i02/>.

Korkmaz, S., Goksuluk, D., & Zararsiz, G. (2014). MVN: An R Package for Assessing Multivariate Normality. *The R Journal, 6*(2), 151–162. <https://journal.r-project.org/archive/2014-2/korkmaz-goksuluk-zararsiz.pdf>.

Jorgensen, T. D., Pornprasertmanit, S., Schoemann, A. M., & Rosseel, Y. (2021). *semTools: Useful tools for structural equation modeling*. R package version 0.5-4. Retrieved from [https://CRAN.R-project.org/package=semTools](https://cran.r-project.org/package=semTools).

**Supplementary material D: MANCOVA assumptions**

| Assumption | Method | Result | Violated | Solution |
| --- | --- | --- | --- | --- |
| Multivariate outliers | Mahalanobis distance | 10 outliers (*MD*=29–52) | Yes | Removed 10 participants |
| Univariate outliers | Cook’s distance | a few influential values | Yes, slightly | Removed 13 participants |
|  | Box-plots | no extreme outliers |  |  |
|  | 1.5x*IQR* rule | 13 outliers |  |  |
| Multivariate normality | Henze-Zirkler test | *p*=.171 | No | –⁠ |
|  | Perspective 3D plot and contour plot | Normal distribution |  |  |
| Univariate normality | Shapiro-Wilk test | *p*<.001 in both subscales | Yes, slightly | Pillai’s Trace (relatively robust) |
|  | Q-Q plots and Histograms | Normal distribution |  |  |
| Multicollinearity | Correlation * | *r*_s_=.70, *p*<.001 | No | –⁠ |
| Relation of covariates with dependent variables | Correlation | HOS & eHSOS did not correlate | Yes | HOS & eHSOS removed from model |
| Linearity | Scatterplots | Linear relationships | No | –⁠ |
| Homogeneity of regression slopes | Interaction between experimental conditions and covariates | Interaction of eHEALS was significant (*p*=.021) | Yes | eHEALS Removed |
| Homogeneity of variances (homoscedasticity) | Levene’s median test | Credibility: *p*=.306  Content: *p*=.019 | Yes, slightly | Pillai’s Trace (MANCOVA) +: Box-cox transformation (ANCOVAs) +  Robust check (non-parametric ANCOVAs) |
|  | Scale-Location plots | Negligible evidence of heteroskedasticity |  |  |
| Homogeneity of covariance | Box’s M-test | *p* =.047 (α=.001) | No | – |
|  | Balanced sample size | *N* per group = 106–124 |  |  |
| Normality of residuals | Shapiro-Wilk test | Credibility: *p*=.028  Content: *p*=.875 | Yes, slightly | Box-cox transformation (ANCOVAs) +  Robust check (non-parametric ANCOVAs) |
|  | Density and Q-Q plots | Normal distribution |  |  |

*note:* * correlation should be lower than .90 (Tabachnick & Fidell, 2012); *p* = *p*-value, *MD* = Mahalanobis distance; *IQR* = interquartile range; *r*_s_ = Spearman's rank correlation coefficient; α = level of significance.

Besides packages described in the main article, we used packages *MVN* (Korkmaz et al., 2014), *psych* (Revelle, 2021), *ggplot2* (Wickham, 2016), and *ggpubr* (Kassambara, 2020).

Korkmaz, S., Goksuluk, D., & Zararsiz, G. (2014). MVN: An R Package for Assessing Multivariate Normality. *The R Journal, 6*(2), 151–162. <https://journal.r-project.org/archive/2014-2/korkmaz-goksuluk-zararsiz.pdf>.

Revelle, W. (2021). *psych: Procedures for Psychological, Psychometric, and Personality Research*. Northwestern University, Evanston, Illinois. R package version 2.1.3, [https://CRAN.R-project.org/package=psych](https://cran.r-project.org/package=psych).

Kassambara, A. (2020). *ggpubr: 'ggplot2' Based Publication Ready Plots*. R package version 0.4.0. [https://CRAN.R-project.org/package=ggpubr](https://cran.r-project.org/package=ggpubr).

Wickham, H. (2016). *ggplot2: Elegant Graphics for Data Analysis*. New York: Springer-Verlag.

Tabachnick, B. G., & Fidell, L.S. (2012). *Using Multivariate Statistics*. 6h Edition. Boston: Person Education.

**Supplementary material E: ANCOVA robustness check**

Non-parametric ANCOVA in the *sm* package (Browman & Azzalini, 2018) found statistically significant differences between groups for both the credibility (*h* = 1.071, *p* < .001) and reliable content (*h* = 1.071, *p* < .001) subscales.

Bowman, A. W., & Azzalini, A. (2018). *R package 'sm': nonparametric smoothing methods* (version 2.2-5.6). http://www.stats.gla.ac.uk/~adrian/sm.
